# Obsessive-compulsive disorder as a comorbidity, risk factor or predictor of dementia

**DOI:** 10.1192/j.eurpsy.2023.523

**Published:** 2023-07-19

**Authors:** A. L. D. C. Ramos, H. Salgado

**Affiliations:** Psychiatry, University Hospital Center of Sao Joao, Oporto, Portugal

## Abstract

**Introduction:**

Although there is sparse evidence about patients with obsessive-compulsive disorder (OCD) that develop dementia, some case reports have suggested an association between these two clinical entities. There have also been descriptions that point to a possible link between late onset OCD and an increased risk or prediction of dementia. Dementia is a common public health problem, exacerbated by the aging of the population, and, without significative improvement in prevention and treatment, its adverse consequences will continue to increase. On the other hand, OCD is a chronic and impairing condition, that typically initiates in adulthood, with variable clinical presentation, impact and prognosis, that can be optimized depending on the therapeutical approach.

**Objectives:**

We propose to review, select and schematize the existing evidence about the association between OCD and dementia. Information about this correlation is considered useful to improve clinical practice in both entities.

**Methods:**

We will analyze the existing literature linking OCD and dementia, considering the articles available in PubMed, published since 2010.

**Results:**

A recent study showed that patients with OCD had increased risk of developing dementia, including Alzheimer’s disease and vascular dementia, compared with control. However, another study on the theme concluded that OCD had no impact in Alzheimer’s disease onset or cognitive impairment. A different study correlated late onset OCD with dementia with Levy bodies, highlighting the importance of testing secondary causes of late onset OCD. There is also a study that correlates OCD with progressive supranuclear palsy, suggesting that dysfunction of the fronto-caudate-thalamus-cerebellum circuit may be involved. Obsessive-compulsive behaviors are also documented symptoms in frontotemporal dementia, existing studies of this overlapping that may elucidate about its neural networks.

**Conclusions:**

Important questions remain unanswered and, to establish an effective correlation between OCD and dementia, clinical investigation in this area should be amplified, mainly with longitudinal studies. Research on the pathogenic and molecular mechanisms potentially common to OCD and dementia may lead to the development of promising therapeutics. Moreover, given its clinical relevance, we consider it pertinent to study the impact of treating properly OCD in reducing the risk of dementia or attenuate its symptoms and progression.

**Disclosure of Interest:**

None Declared

